# Supplementary material for: The Therapeutic Potential of Low-Intensity Pulsed Ultrasound in Enhancing Gallbladder Function and Reducing Inflammation in Cholesterol Gallstone Disease
Source: Bioengineering (Basel). 2025 Jan 4;12(1):34. doi: 10.3390/bioengineering12010034 (PMC11762117; doi:10.3390/bioengineering12010034)
Supplement: Supplementary file 1 [file bioengineering-12-00034-s001.zip › normality and homogeneity of variance.pdf]

The data were tested by Graphad 9.0 for normality and homogeneity of variance.Here is an example for normality and homogeneity of variance test of gallbladder emptting rates(GBEF).

	Normality and Lognormality Tests<br>Tabular results	A<br>NC	B<br>GS	C<br>LIPUS (-)	D<br>LIPUS (+)
1	**Test for normal distribution**				
2	**Shapiro-Wilk test**				
3	W	0.9196	0.9252	0.8576	0.8718
4	P value	0.3885	0.4371	0.0902	0.1287
5	Passed normality test (alpha=0.05)'	Yes	Yes	Yes	Yes
6	P value summary	ns	ns	ns	ns
7					
8	**Number of values**	9	9	9	9

consistent with normal distribution

	Ordinary one-way ANOVA<br>ANOVA results					
4	**ANOVA summary**					
5	F	32.31				
6	P value	<0.0001				
7	P value summary	****				
8	Significant diff. among means (P < 0.05)'	Yes				
9	R squared	0.7518				
10						
11	**Brown-Forsythe test**					
12	F (DFn, DFd)	0.1542 (3, 32)				
13	P value	0.9262				
14	P value summary	ns				
15	Are SDs significantly different (P < 0.05)?	No				
16						
17	**Bartlett's test**					
18	Bartlett's statistic (corrected)	2.273				
19	P value	0.5178				
20	P value summary	ns				
21	Are SDs significantly different (P < 0.05)?	No				
22						
23	**ANOVA table**	**SS**	**DF**	**MS**	**F (DFn, DFd)**	**P value**
24	Treatment (between columns)	28726	3	9575	F (3, 32) = 32.31	P<0.0001
25	Residual (within columns)	9484	32	296.4		
26	Total	38210	35			
27						
28	**Data summary**					
29	Number of treatments (columns)	4				
30	Number of values (total)	36				

The P-value of the homogeneity of variance test is $>$ 0.05， the variance is homogeneous
