# Supplementary figures and images for: The Therapeutic Potential of Low-Intensity Pulsed Ultrasound in Enhancing Gallbladder Function and Reducing Inflammation in Cholesterol Gallstone Disease
Source: Bioengineering (Basel). 2025 Jan 4;12(1):34. doi: 10.3390/bioengineering12010034 (PMC11762117; doi:10.3390/bioengineering12010034)

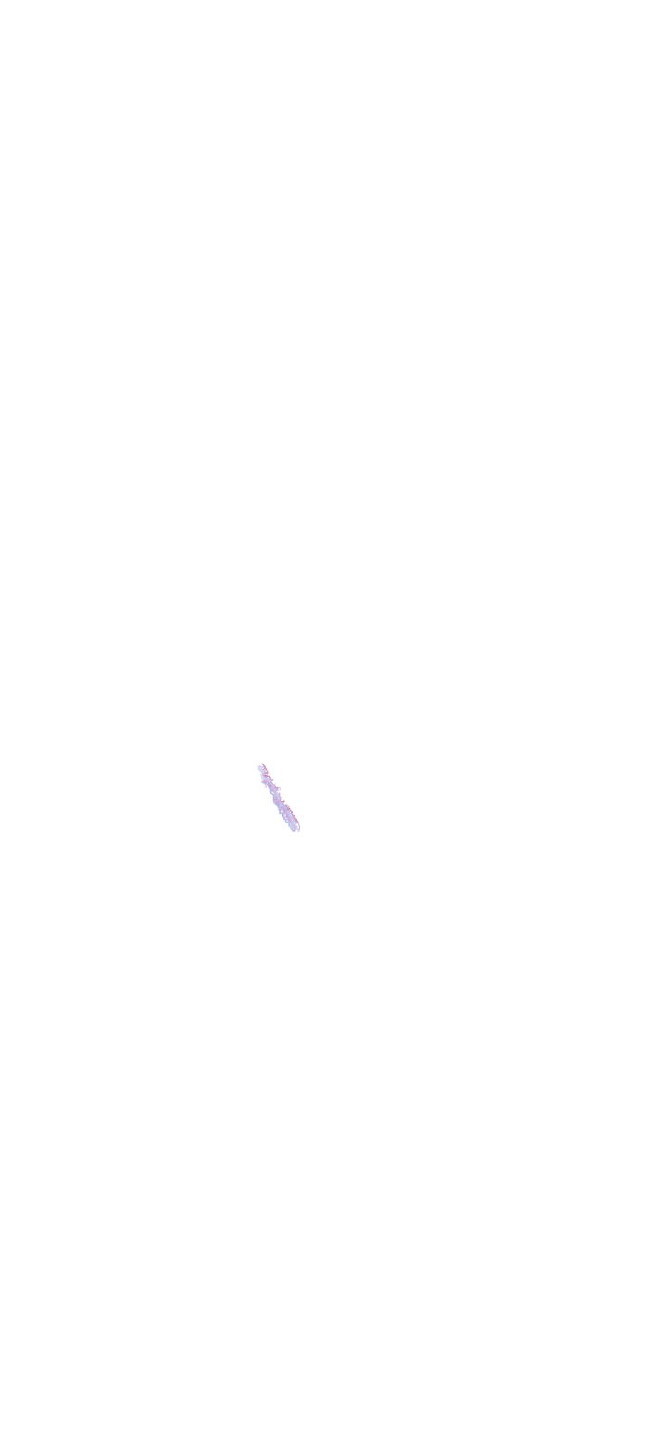

Supplement: Supplementary file 1 [file bioengineering-12-00034-s001.zip › LIPUS(+) histology images(HE and MASSON)/LIPUS(+)1 masson.mrxs]

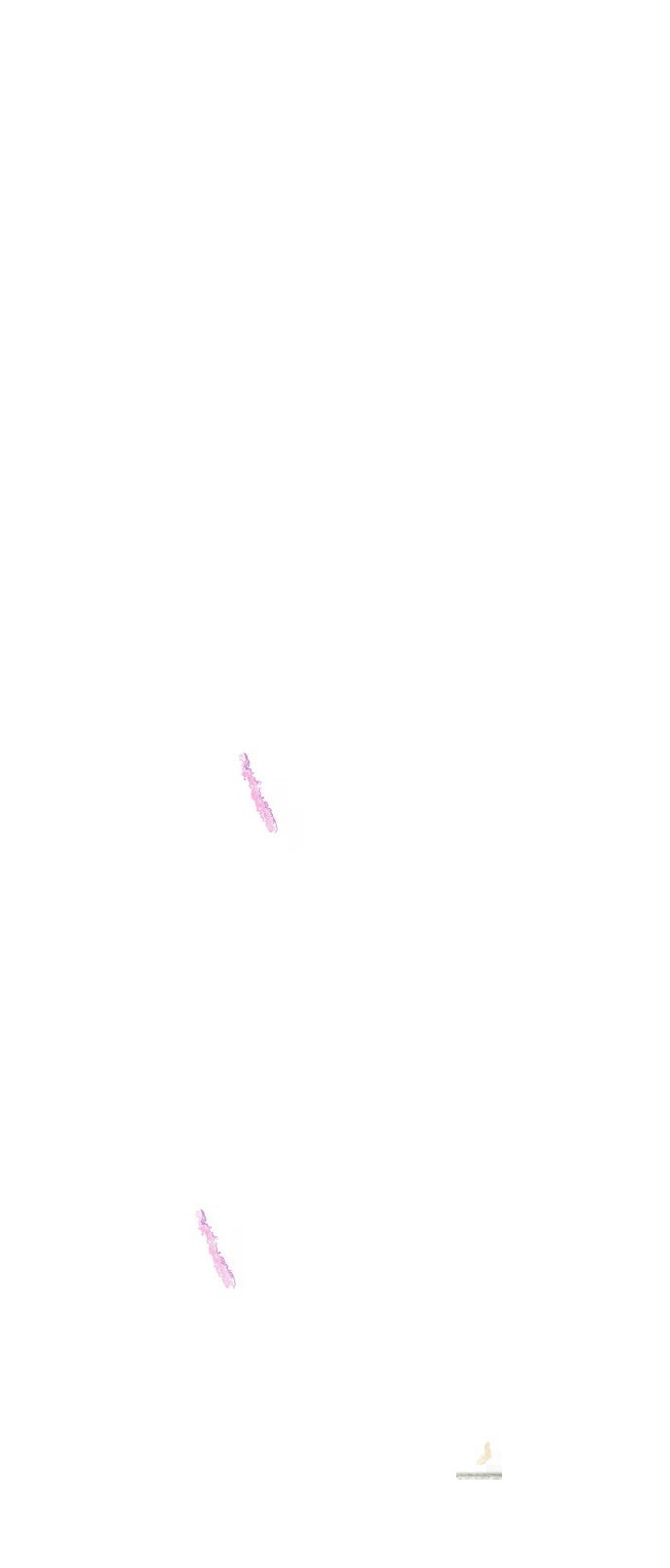

Supplement: Supplementary file 1 [file bioengineering-12-00034-s001.zip › LIPUS(+) histology images(HE and MASSON)/LIPUS(+)1 HE.mrxs]

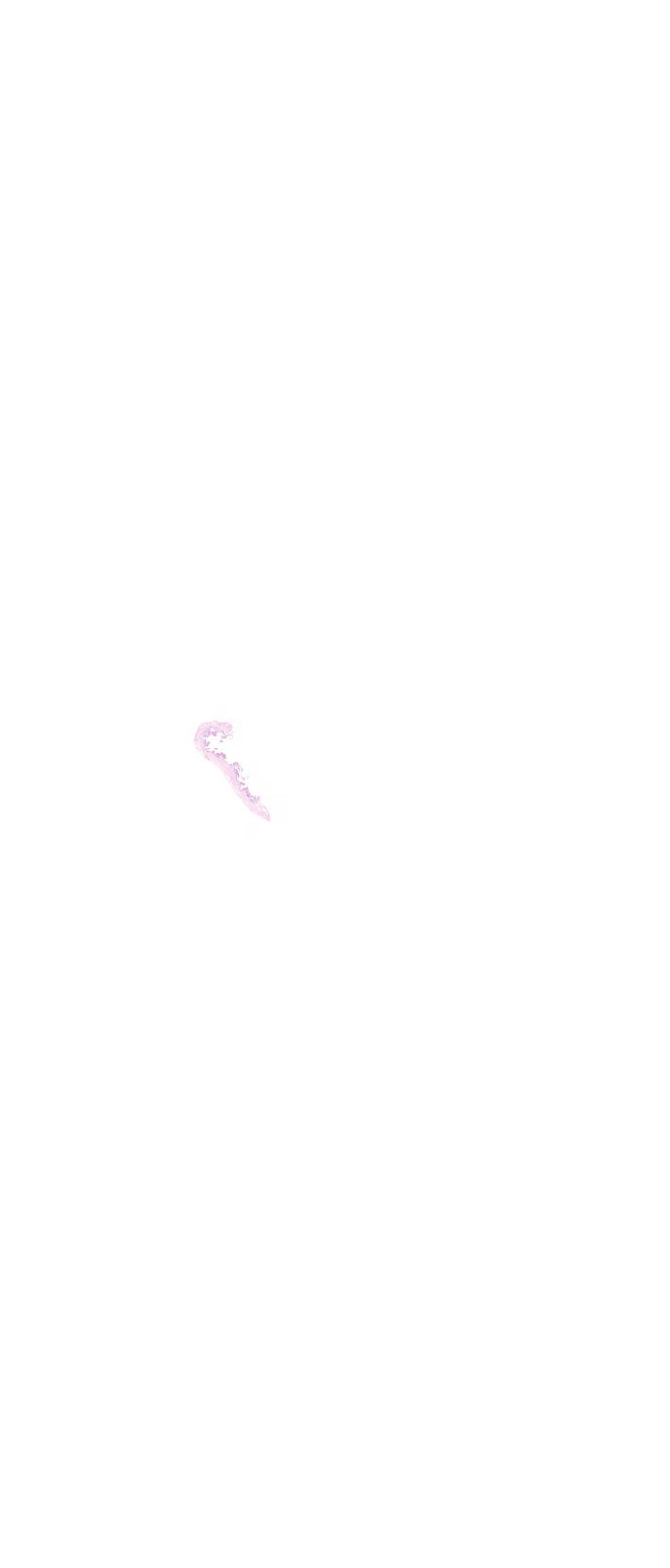

Supplement: Supplementary file 1 [file bioengineering-12-00034-s001.zip › LIPUS(-) histology images(HE and MASSON)/GS2- α-HE.mrxs]

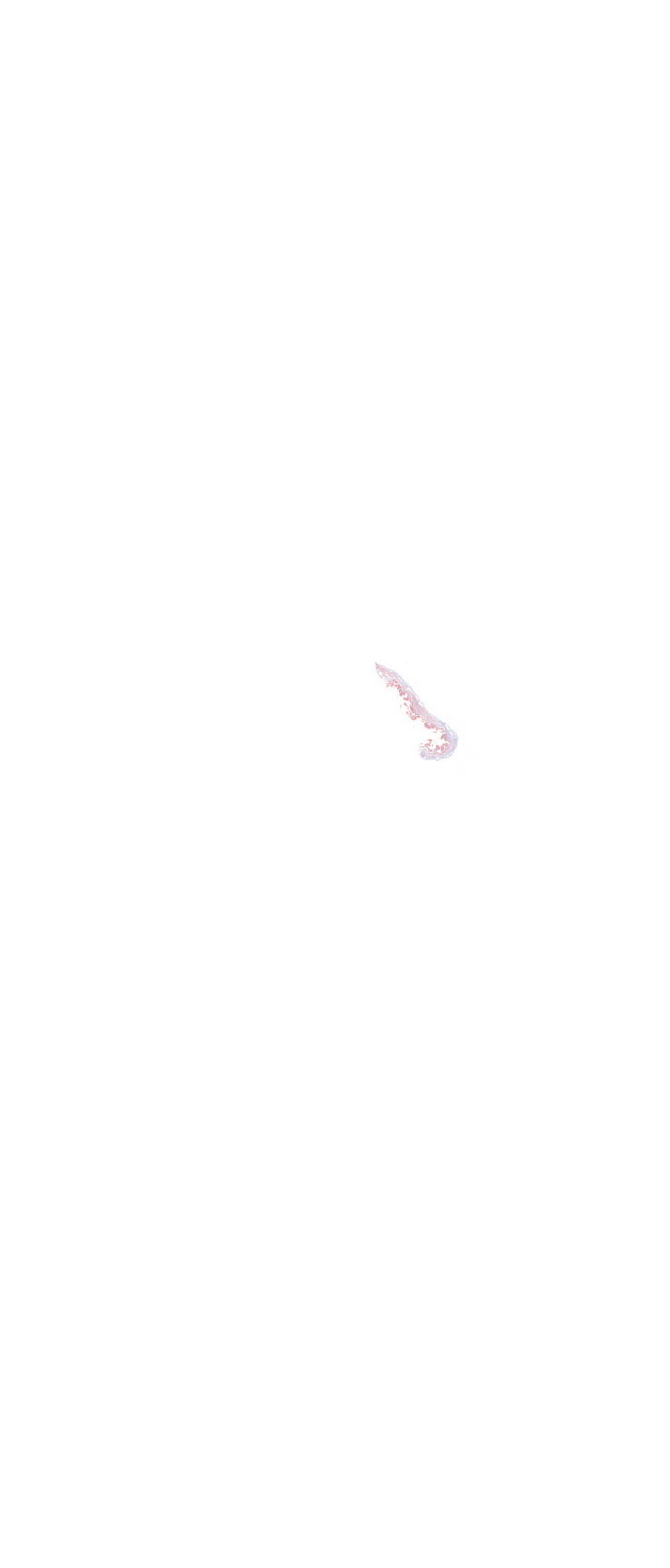

Supplement: Supplementary file 1 [file bioengineering-12-00034-s001.zip › LIPUS(-) histology images(HE and MASSON)/GS2- α-masson.mrxs]
